# Supplementary material for: Gut microbiome variation in juvenile blue tits in a European urban mosaic
Source: Sci Rep. 2025 Nov 11;15:39474. doi: 10.1038/s41598-025-23005-y (PMC12606331; doi:10.1038/s41598-025-23005-y)
Supplement: Supplementary file 1 — Supplementary Information 1. [file 41598_2025_23005_MOESM1_ESM.pdf]

Supplementary Information for:

"Gut microbiome variation in juvenile blue tits in a European urban mosaic."

Lena Fus[1,*], Sebastian Jünemann[2,3], Irene Di Lecce[1], Joanna Sudyka[4], Marta Szulkin[1,+], Öncü Maraci[5,6,+]

[1] Institute of Evolutionary Biology, Biological and Chemical Research Centre, Faculty of Biology, University of Warsaw, Warsaw, Poland

[2] Faculty of Technology, Bielefeld University, Universitätsstrasse 25, 33615, Bielefeld, Germany

[3] Institute for Bio- and Geosciences, IBG-5, Research Center Jülich, Bielefeld University, Universitätsstraße 27, 33615, Bielefeld, Germany

[4] Institute of Environmental Sciences, Jagiellonian University, Kraków, Poland

[5] Department of Behavioural Ecology, Bielefeld University, Konsequenz 45, 33619 Bielefeld, Germany.

[6] Joint Institute for Individualisation in a Changing Environment (JICE), University of Münster and Bielefeld University, Münster, Germany.

***Correspondence:** Lena Fus

E-mail: lenafus.lf@gmail.com

[+] These authors contributed equally.

Supplementary Table 1. Results of differential abundance of microbial families between years. Both listed families were more abundant in 2018. Only families with statistically significant differences are shown. 2 out of 344 families were found to be differentially abundant between years.

padj	p	Phylum	Family
0.0084594	0.0000379	Verrucomicrobiota	Chlamydiales unclassified
0.0212351	0.0001904	Firmicutes	*Streptococcaceae*

Supplementary Table 2a. Linear models: alpha diversity between two types of cavities and two years.

Predictors	Response variable	Est	CI	p
year (2019 vs. 2018)	Chao1	-13.39	-92.19 - 65.41	0.731
	Shannon	0.22	-0.93 - 1.38	0.699
	Faith's PD	-0.79	-5.09 - 3.5	0.709
cavity type (nestbox vs. natural)	Chao1	40.97	-29.85 - 111.78	0.246
	**Shannon**	**1.3**	**0.26 - 2.34**	**0.016**
	Faith's PD	2.65	-1.21 - 6.51	0.171
year * cavity type	Chao1	-81.72	-201.43 - 37.98	0.173
	**Shannon**	**-2.38**	**-4.13 - -0.62**	**0.01**
	Faith's PD	-5.66	-12.18 - 0.87	0.087

Supplementary Table 2b. Results of post-hoc tests (year and cavity type).

Contrast		Index	Est	SE	p
BOX-2018	NAT-2018	Shannon	1.301	0.507	0.071
		Faith's PD	0.4475	0.284	0.4075
**BOX-2018**	**BOX-2019**	**Shannon**	**2.156**	**0.646**	**0.0118**
		**Faith's PD**	**1.0972**	**0.361**	**0.0244**
BOX-2018	NAT-2019	Shannon	1.08	0.507	0.1681
		Faith's PD	0.5221	0.284	0.2763
NAT-2018	BOX-2019	Shannon	0.855	0.692	0.6096
		Faith's PD	0.6497	0.387	0.3526
NAT-2018	NAT-2019	Shannon	-0.221	0.565	0.9794
		Faith's PD	0.0746	0.316	0.9953
BOX-2019	NAT-2019	Shannon	-1.075	0.692	0.4192
		Faith's PD	-0.5751	0.387	0.4584

Supplementary Table 2c. Results of PERMANOVA tests for beta diversity between cavity types.

Fixed effect	Response variable	Pseudo-F	p
year	Jaccard	1.11781	0.0522
	**Bray-Curtis**	**1.2337**	**0.0377**
	UniFrac	1.15088	0.1321
	Weighted UniFrac	1.0815	0.3145
cavity type	Jaccard	1.0598	0.1642
	Bray-Curtis	1.0923	0.1867
	UniFrac	1.15587	0.1345
	Weighted UniFrac	1.1812	0.2491

Supplementary Table 3. Sample size and the distribution of ISA among the sampling sites.

Habitat type	Cavity type	Initial N	Final N	Min	Max	Mean	SD	Median
peri-urban village	nest box	6	6	0.41	5.53	1.89	1.85	1.36
natural forest	nest box	12	11	0.00	0.00	0.00	0.00	0.00
urban forest	nest box	19	17	0.00	4.09	0.80	1.35	0.00
	natural cavity	17	16	NA	NA	NA	NA	NA
residential area I	nest box	4	4	36.97	42.95	40.38	2.60	40.79
residential area II	nest box	3	2	37.74	41.00	39.37	2.30	39.37
urban woodland I	nest box	17	16	0.00	32.13	5.89	8.99	0.60
urban woodland II	nest box	3	3	9.11	20.25	14.27	5.61	13.46
urban park	nest box	36	30	0.16	43.16	10.89	10.68	6.20
office area	nest box	2	2	42.04	55.81	48.92	9.73	48.92
	total:	119	**107**					

Supplementary Figure 1. Pearson's correlation coefficients among ISA and environmental variables.

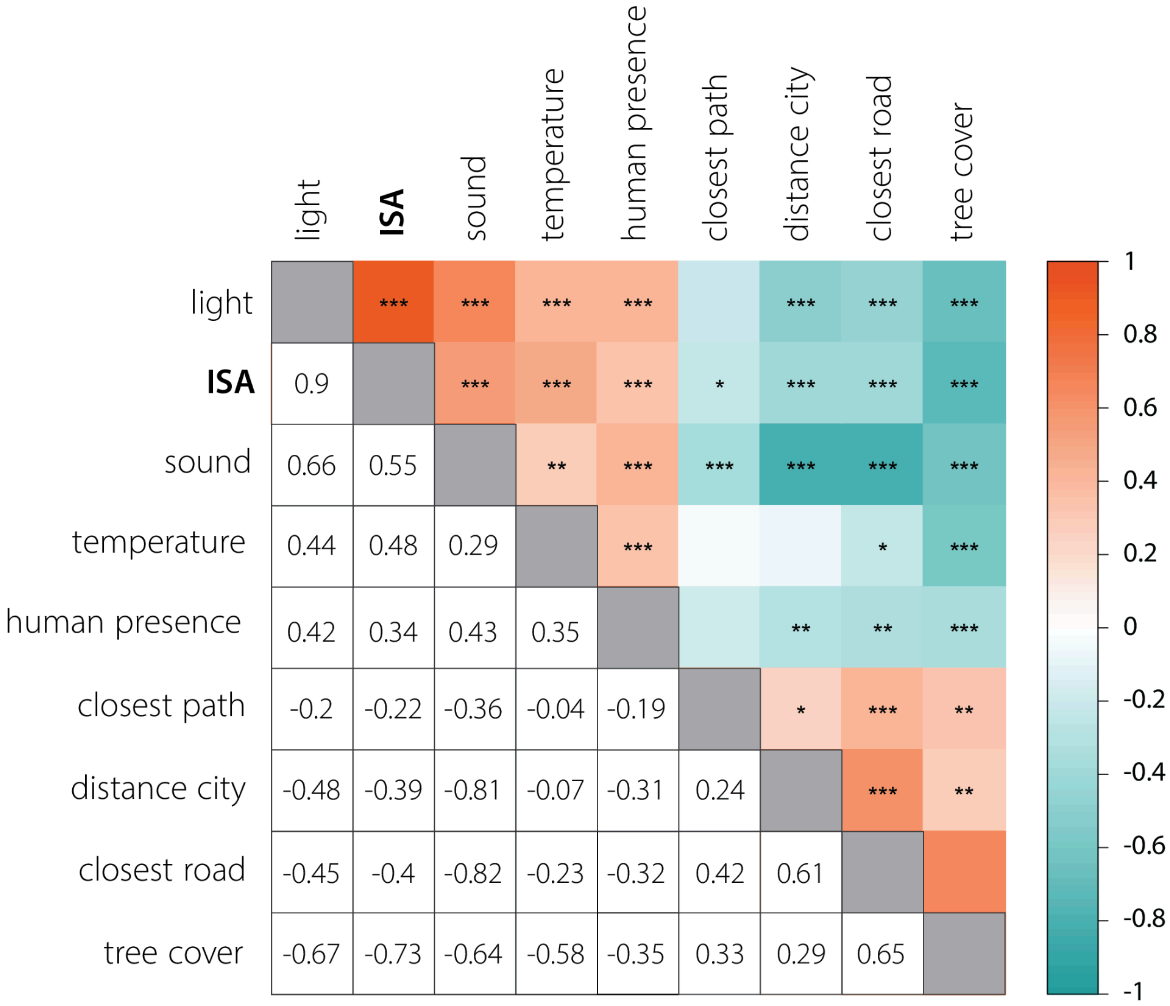

Supplementary Text 1. Environmental and spatial variables used in the study.

1. **variables collected on the ground:**

(a) human presence, derived by quantifying all humans and dogs within a 15 m radius around each nest box (Corsini et al., 2017),
(b) sound pollution, obtained after averaging recordings on the DbC scale using hand-held sound level metres over four days throughout the field season (Szulkin et al., 2020), and
(c) temperature, measured with Thermocrone ibuttons DS1921G set in 2018 from April 24 until June 30 (Szulkin et al., 2020).

2. **variables extrapolated from digital photography and satellite imagery:**

(a) tree cover density, derived from a map downloaded from Copernicus Land Monitoring Services, and
(b) light pollution, extrapolated from night-time digital photographic images shot on 08/10/2015 by astronauts from the International Space Station (Kyba et al., 2015).

Additionally, **spatial variables** including distance to the city centre (Palace of Culture and Science), closest road, and closest path, all measured in metres in QGIS (Corsini et al., 2017).

References:

Corsini, M., Dubiec, A., Marrot, P., & Szulkin, M. (2017). Humans and tits in the city: Quantifying the effects of human presence on great tit and blue tit reproductive trait variation. Frontiers in Ecology and Evolution, 5, 82.

Kyba, C., Garz, S., Kuechly, H., De Miguel, A. S., Zamorano, J., Fischer, J., & Hölker, F. (2015). High-resolution imagery of earth at night: New sources, opportunities and challenges. Remote sensing, 7(1), 1-23.

Szulkin, M., Garroway, C. J., Corsini, M., Kotarba, A. Z., Dominoni, D., & Szulkin, M. (2020). How to quantify urbanization when testing for urban evolution. Urban evolutionary biology, 13(1), 1861-1876.
